# Immune microenvironment of experimental rat C6 gliomas resembles human glioblastomas

**DOI:** 10.1038/s41598-017-17752-w

**Published:** 2017-12-14

**Authors:** Anna Gieryng, Dominika Pszczolkowska, Katarzyna Bocian, Michal Dabrowski, Wenson David Rajan, Michal Kloss, Jakub Mieczkowski, Bozena Kaminska

**Affiliations:** 1 Laboratory of Molecular Neurobiology, Neurobiology Center, Nencki Institute of Experimental Biology of Polish Academy of Sciences, Warszawa, Poland; 2 Laboratory of Bioinformatics, Neurobiology Center, Nencki Institute of Experimental Biology of Polish Academy of Sciences, Warszawa, Poland

## Abstract

Glioblastoma (GBM) is the most aggressive primary brain tumor, with ineffective anti-tumor responses and a poor prognosis despite aggressive treatments. GBM immune microenvironment is heterogenous  and activation of specific immune populations in GBM is not fully characterized. Reliable animal models are critical for defining mechanisms of anti-tumor immunity. First we analyzed the immune subpopulations present in rat C6 gliomas. Using flow cytometry we determined kinetics of infiltration of myeloid cells and T lymphocytes into glioma-bearing brains. We found significant increases of the amoeboid, pro-tumorigenic microglia/macrophages, T helper (Th) and T regulatory (Treg) cells in tumor-bearing brains, and rare infiltrating T cytotoxic (Tc) cells. Transcriptomic analyses of glioma-bearing hemispheres revealed overexpression of invasion and immunosuppression-related genes, reflecting the immunosuppressive microenvironment. Microglia, sorted as CD11b^+^CD45^low^ cells from gliomas, displayed the pro-invasive and immunosuppressive type of activation. Accumulation of Th and Treg cells combined with the reduced presence of Tc lymphocytes in rat gliomas may result in the lack of effective anti–tumor responses. Transcriptional profiles of CD11b^+^ cells and composition of immune infiltrates in C6 gliomas indicate that rat C6 gliomas employ similar immune system evasion strategies as human GBMs.

## Introduction

Tumor cells and their products are capable of regulating and altering gene expression in non-tumor cells within or infiltrating into the microenvironment, thereby shaping their phenotype. Although various immune effector cells are recruited to the tumor site, their anti-tumor functions are down-regulated, largely in response to tumor-derived signals. Numerous immunosuppressive networks operate in malignant tumors^1–4^. Reliable animal models are critical for defining tumor immunity mechanisms.

Glioblastoma (GBM) is the most common and aggressive primary brain tumor. Due to frequent dysfunctions of tumor suppressors and oncogenes, diffusive growth and resistance to standard therapies, the median survival is approximately 14 months and the treatment remains mainly palliative^5–7^. Histopathological and flow cytometry analyses of human gliomas revealed infiltration of GBMs with numerous immune cells: resident (microglia) and peripheral macrophages (collectively called glioma-associated microglia/macrophages, GAMs), granulocytes, myeloid-derived suppressor cells (MDSCs) and T lymphocytes (for reviews^1,8,9^). Intratumoral densities of glioma-associated microglia/macrophages (GAMs) and MDSCs were correlated with the histological grade of gliomas, immunosuppression and patient’s survival^10–12^. A recent pan-cancer analysis using mRNA sequencing data from The Cancer Genome Atlas for 11 tumor types representing 3485 tumors revealed that macrophage signatures predicted worse survival in GBM^13^. Although GAMs have a few innate immune functions intact, this is not sufficient to initiate effective immune responses^14,15^. Moreover, GAMs support tumor invasion^16–19^ and release immunosuppressive cytokines and chemokines. MDSCs inhibit cytotoxic responses mediated by natural killer cells, and block the activation of tumor-reactive CD4 + T helper (CD4 + Th) cells and CD8 + T cytotoxic (CD8 + Tc) cells^12,20^. The presence of regulatory T cells may also contribute to the lack of effective immune responses against malignant gliomas^21^.

Tissue macrophages, depending on a stimulus, activate inflammatory responses or cytoprotective, immunosuppressive responses typical for macrophages involved in tissue repair and inflammation resolution^22^. Studies attempting to dissect a functional phenotype of human GAMs demonstrated conflicting results indicating either a non-inflammatory, pro-tumorigenic phenotype^10^, a mixed inflammatory/anti-tumor phenotype^23^ or M0 phenotype^24^. Among leukocytes, mostly CD4^+^ Th, CD4^+^CD25^+^FoxP3^+^ T regulatory (Treg) and CD8^+^ Tc subsets were detected in GBMs^20,21,25–27^. Accumulation of CD4^+^Th and CD4^+^Treg subsets in GBMs was correlated with the worst prognosis^28^. Increased expression *TGF-β* (transforming growth factor β) and *IL-10* (interleukin-10) in GBMs may result in inhibition of Tc activation/proliferation and differentiation of naïve T cells into Treg^25^.

Animal models are still instrumental for providing ‘proof of concept” and appropriate rodent glioma models that recapitulate innate and adaptive immune responses occurring in human GBMs would be desirable. Rat C6, 9 L and T9 gliomas were induced by repeated injections of methylnitrosourea to pregnant rats. C6 glioma cells which typically grow in an outbred, not syngeneic Wistar rat and have potential to evoke an alloimmune response^29^, are commonly used as a glioma model in pharmacological studies. *In vivo* magnetic resonance imaging and angiography studies showed that similarity of C6 gliomas to human malignant gliomas is better than other models^30^. In the present study, we analyzed immune heterogeneity in rat C6 gliomas and kinetics of immune cell infiltration of GAMs, MDSCs and T cell subpopulations. We found overexpression of invasion and immunosuppression-related genes in glioma-bearing hemispheres and in tumor infiltrating microglia. Characteristics of immune infiltrates, gene expression profiles and well known histopathological similarities of C6 gliomas to human GBMs, led to conclusion that the rat C6 gliomas employ similar immune evasion strategies as human mesenchymal GBMs.

## Material and Methods

### Animals

Male Wistar rats (2–3 months old, body weigh 250–300 g at the beginning of the study) were housed with free access to food and water, on a 12 h/12 h day and night cycle. All efforts have been made to minimize the number of animals and animal suffering. All research protocols conformed to the Guidelines for the Care and Use of Laboratory Animals (European and national regulations 2010/63/UE September 22, 2010 and Dz. Urz. UE L 276/20.10.2010, respectively). Animals were decapitated by a qualified researcher. The First Warsaw Local Ethics Committee for Animal Experimentation approved the study (the protocol 262/2012 from 15/03/2012).

### Cell culture

C6 rat glioma cells from the American Type Culture Collection were cultured in Dulbecco’s modified Eagle medium (DMEM) containing 10% NCS, 100 U/ml penicillin and 100 µg/ml streptomycin (PenStrep) (GIBCO, Invitrogen, Paisley, Scotland, UK). Cell cultures were maintained at 37 °C in a humidified chamber of 95% air/5% CO_2_. For implantation cells were trypsinized (0.25% trypsin) for 3 min, washed, centrifugated for 10 min at 1000 rpm, and 1 × 10^6^ cells were re-suspended in 50 µl in DMEM and kept on ice.

### Stereotactic implantation glioma cells

Rats were isoflurane-anaesthetized and placed in a stereotaxic instrument (Stoelting, Hertfordshire, UK). The stereotaxic coordinates of the intracranial injection were AP =  + 1.6 mm to bregma, ML = −2 mm lateral to bregma, and DV = −6 mm ventral. Animals received a single microinjection of 5 × 10^4^ C6 rat glioma cells (in 2.5 µl) or DMEM (2.5 µl) into the right stratum using 10 µl syringe (Hamilton, Bonaduz, Switzerland), at a constant rate of 0.5 µl/min at the day 0 of the study. Rats were divided into 3 groups: untreated animals (N = 14), sham operated group receiving DMEM (N = 12) or C6 rat glioma group (N = 31), and sacrificed at day 8^th^, 14^th^, 15^th^, 21^st^ after injection.

### Isolation of immune cells from brain hemispheres and blood

In the first set of experiments naive, sham-operated and glioma bearing rats were sacrificed at day 8^th^, 14^th^, 21^st^ after cell inoculations. In further experiments rats implanted with C6 cells or medium were sacrificed at day 21^st^ and perfused with cold Phosphate-Buffered Saline (PBS) (Invitrogen, Paisley, UK). Immediately after perfusion, brains were isolated and stored in HBSS (without calcium, magnesium) (Invitrogen, Paisley, UK). Mechanical and enzymatical tissue dissociation of brain hemispheres was performed with the use of gentle MACS Dissociator, Neural Tissue Dissociation Kit (P) in MACS C Tubes (MACS, Miltenyi Biotec GmbH, Bergish Glabdach, Germany) according to manufacturer’s instructions. Myelin was removed using Percoll (GE Healthcare Bio-Sciences AB, Uppsala, Sweden) density gradient centrifugation. Briefly, a single suspension of cells was mixed with the Percoll solution and centrifugated at 2100 rpm for 20 min, at 4 °C. Collected cells were used for flow cytometry analyses.

From the same animals, peripheral blood mononuclear cells (PBMCs) were isolated from heparinized blood by density-gradient centrifugation over Gradisol L (Aqua Medica, Lodz, Poland). PBMC were washed with cold PBS and re-suspended as 1 × 10^6^ cells/100 µl in Staining Buffer (BD Bioscences, Becton, Dickinson and Company, New Jersey, USA) for future analyses.

### Flow cytometry studies

Specific immune cell populations were identified by fluorescence-activated cell sorting (FACS) based on the expression of characteristic markers: microglia (CD11b^+^CD45^low^), macrophages (CD11b^+^CD45^high^) and leukocytes (CD45^high^), MDSCs (CD11b^+^GR1^+^), Th (CD4^+^), Tc (CD8a^+^) and Treg lymphocytes (intracellular FOXP3^+^).

Cells were stained immediately after Percoll gradient separation, using antibodies with the following specificities: FITC Mouse Anti-Rat CD11b (clone WT.5), FITC Mouse IgA Kappa Isotype Control, PE Mouse Anti-Rat CD45 clone (OX-1), PE Mouse IgG1 Kappa Isotype Control, PE Mouse Anti-Rat Gr1 (clone RP-1), PE Mouse IgG2a Kappa Isotype Control (from BD Pharmingen, BD Biosciences), FITC Anti-Rat CD4 (clone W3/25), FITC Mouse IgG1, κ Isotype Control, PerCP Anti-rat CD8a (clone OX-8) and PerCP Mouse IgG1, κ Isotype Control; intracellular staining of FOXP3 was performed with PE Anti-mouse/rat/human FOXP3 (clone 150D), PE Mouse IgG1, Kappa Isotype Control and FOXP3 Fix/Transcription Factor Staining Buffer Set (BioLegend, San Diego, USA) according to the manufacturer’s instructions. IgG1 antibodies conjugated with the respective fluorochromes were used to determine background staining and served as isotype controls.

Briefly, 1 × 10^6^ cells (in 100 μL FACS Staining Buffer) were incubated with antibodies for 30 min on ice in a 5 mL polypropylene round-bottom tube (BD Biosciences, San Diego, CA) to prevent adherence of myeloid cells. Subsequently, cells were washed in FACS Staining Buffer, centrifugated at 1400 rpm for 6 min and re-suspended in 100 μL FACS buffer. Flow cytometric analysis was performed with identical instrument settings on a FACS Calibur machine using Cell Quest software (BD Biosciences). The results were based on the analysis of at least 5 × 10^4^ cells (and 1 × 10^6^ cells for intracellular FOXP3 staining), and are shown as a percentage of positively labeled cells among all gated cells.

### Immunohistochemistry

The animals were sacrificed at the day 15^th^ after glioma cell implantation and perfused with 4% paraformaldehyde in PBS. Brains were removed, post-fixed for 48 h in the same fixative solution and placed in cryprotective solution of 30% sucrose in PBS at 4 °C. Tissue was frozen in Tissue Freezing Medium (Jung; Nussloch, Germany) and cut in 12 µm coronal sections using a cryostat.

Sections were stained using Iba-1-specific antibody (Wako, Osaka, Japan) in order to visualize microglia/macrophages infiltrating the tumor. Anti-Arginase 1 antibody (Santa Cruz Biotechnology, Santa Cruz, CA, USA) was used to determine functional phenotype. Slides were treated with 1% SDS in PBS for 5 minutes for membrane permeabilization. To prevent unspecific binding, sections were incubated with a blocking solution (10% donkey serum in PBS) for 1 hour at room temperature and then stained with a cocktail of rabbit anti-Iba1 antibody (1:100) and goat anti-Arg1 antibody (1:100) in 3% donkey serum at 4 °C overnight, followed by 2 h incubation with the Alexa Fluor 488-conjugated donkey anti-goat antibody (1:1000, Invitrogen) and Alexa Fluor 555-conjugated donkey anti-rabbit (1:1000, Invitrogen). Incubation was followed by labeling with DAPI in PBS (1:1000, Sigma-Aldrich, Saint Louis, MO) and closing slides with Fluorescent Mounting Medium (Dako North America Inc., Carpinteria, CA, USA). Fluorescent images were acquired using a Leica DM4000B microscope.

### Microarray analysis of brain tissues and isolated CD11b^+^CD45^low^ cells

For transcriptomic analyses of bulk tumors, total RNA was isolated from tumor bearing hemispheres or brain hemispheres of naive rats sacrificed at day 21^st^ after glioma implantation. Immediately after decapitation, hemispheres were isolated and frozen in liquid nitrogen and kept in −80 °C until further analysis. Samples were homogenized in TRI Reagent (Sigma-Aldrich, Saint Louis, MO) (130–150 mg of tissue in 1 ml) using PRO200 homogenizer (Bioeko, Germany) according to manufacturer’s protocol. RNA quality and purity was determined using NanoDrop (Thermo Scientific, Germany). Samples were purified using High Pure RNA Isolation Kit (Roche, Germany) according to manufacturer’s protocol to remove traces of reagents used during isolation. Amount and quality of isolated RNAs were determined by capillary electrophoresis with RNA 6000 Nano Kit (Agilent Technologies) and Bioanalyzer 2100 (Thermo Scientific, Germany). Microarray experiments were performed with 100 ng of total RNA as a template. Whole-genome amplification procedure was performed with a GeneAtlas WT Expression Kit according to the manufacturer’s User Guide for the GeneAtlas Personal Microarray System (Affymetrix, Santa Clara, CA, USA). Fragmented and labeled cDNA was hybridized to the Affymetrix Rat Gene 2.1 ST Array Strip.

For global analysis of gene expression in microglia, CD11b^+^CD45^low^ cells were isolated from tumor bearing hemispheres or brain hemispheres of naive rats sacrificed at day 14^st^ after glioma implantation. Cells were stained with monoclonal antibodies: FITC Mouse Anti-Rat CD11b (clone WT.5) and PE Mouse Anti-Rat CD45 (clone OX-1) (BD Pharmingen) to distinguish between resident microglia (CD11b^+^CD45^low^) and infiltrating macrophages (CD11b^+^CD45^high^). Flow cytometry sorting was performed at a BD FACSAria II platform (BD Bioscience). Total RNA was isolated from CD11b^+^CD45^low^ FACS-sorted cells using High Pure RNA Isolation Kit (Roche; Mannheim, Germany). RNA quality and purity was analyzed with nanophotometer and RNA 6000 Nano Kit (Agilent Technologies) and Bioanalyzer 2100 (Thermo Scientific).

Sample processing was performed at the Cambridge Genomic Services, Department of Pathology, University of Cambridge, UK. Fragmented and labeled cDNA was hybridized to the Affymetrix Rat Gene 2.1 ST Array Strip (RaGene-2.1_st.) (Affymetrix, Santa Clara, CA, USA). The Affymetrix Transcriptome Analysis Console was used to perform the differential analysis, on the exon level data.

### Bioinformatic analysis

The microarray data were preprocessed with R Bioconductor oligo rma function using the transcript (core) option and annotated to the genes with the getNatAffx function. Only the profiles uniquely mapping to genes were then averaged for each gene. Welch’s t-test and data visualization as heatmaps were performed in python using scipy and matplotlib libraries’ functions.

Gene Ontology terms associated with changes in gene expression were identified using RankGOstat^31^. The lists of gene symbols, together with a log2-change in expression between the compared groups were used as the input files. Default options (Wilcoxon Signed Rank test, Benjamini False Discovery Rate correction for multiple testing) were used, with the RGD database chosen as the source of GO annotations and the analysis was restricted to the “biological process” ontology. The result files were saved, parsed and converted to graphics using local scripts.

For comparison with human GBM transcriptomic profiles, RNA-seq profiles (level 3) of GBM and control samples were downloaded from TCGA website. GBM subtypes (Proneural, Neural, Classical and Mesenchymal) were assigned using the published, 840-gene signature^32^ and one-sample Kolmogorov-Smirnoff test. Rat genes were mapped to their human orthologues using 87 release of Ensembl annotation. The list of orthologues contained 660 out of 840 genes included in the signature. Spearman’s correlation coefficients between fold-changes (tumor/normal) of the 660 genes were used to compare C6 model and GBM subtypes.

### Accession numbers

All original microarray data were deposited at the ArrayExpress accession number: E-MTAB-5049; title: Microarray gene expression profiling of the whole brain hemisphere in the rats with C6-glioma tumor, and E-MTAB-5050; title: Microarray gene expression profiling of microglia from control and C6 glioma tumor-bearing brain hemisphere in the rat.

### Statistical analysis

All values are presented as mean ± standard error of the mean (SEM); differences between animals were considered statistically significant when *p* < 0.05 using the t test followed by Mann Whitney U test. Data were analyzed with the statistical analysis package StatGraphics 5.0 (STSC Inc., Rockville, MD, USA).

## Results

### Kinetics of immune cell accumulation in intracranial C6 gliomas

**Figure 1:**
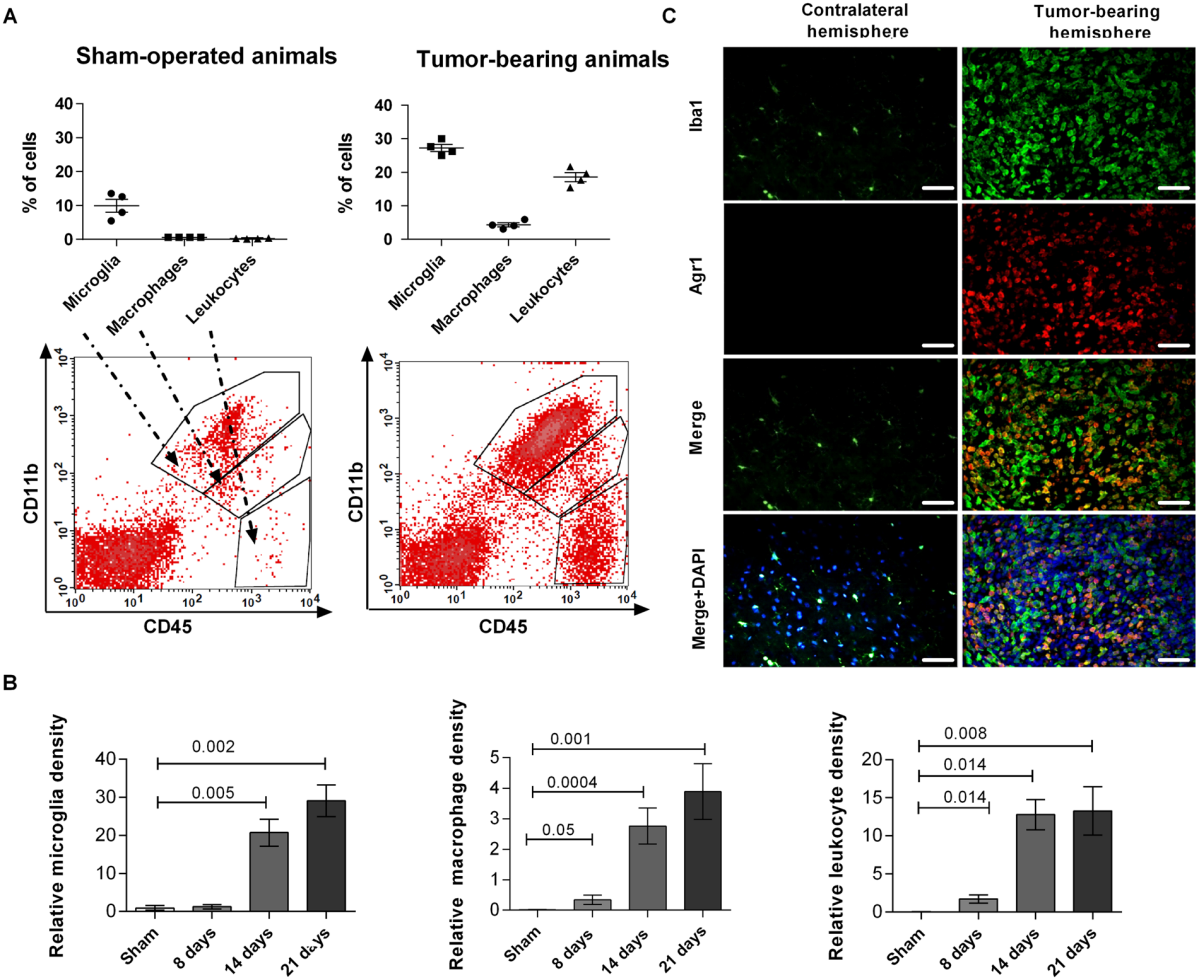
Evaluation of the content of microglia, blood-derived macrophages and leukocytes in C6 rat gliomas. (**A**) Percentages of microglia, blood-derived macrophages and leukocytes in brains of sham-operated and C6 rat glioma bearing animals at day 21^st^ after implantation. Lower panel shows gating strategy and representative results of FACS analysis. Quadrant gates were drawn on three cell subpopulations based on differences in the surface expression of CD11b and CD45 antigens: microglia (CD11b^+^CD45^low^), blood-derived macrophages (CD11b^+^CD45^high^) and leukocytes (CD11b^-^CD45^high^). Leukocytes CD11b^-^CD45^high^ and CD11b^+^CD45^high^ invading macrophages/monocytes were mostly absent in sham-operated brain samples. (**B**) Kinetics of accumulation of microglia, peripheral macrophages and leukocytes within the tumor. Changes in cell type content at 8^th^, 14^th^ and 21^st^ day after implantation were calculated as content of each subpopulations in glioma bearing brains related to its content in sham-operated animals (N = 4–6 per time point). (**C**) Double staining of Iba1 + and Arginase 1 shows accumulation and the pro-tumorigenic activation of GAMs only in tumor-bearing hemispheres (N = 3–5 rats per group).

Previous studies established a flow cytometry phenotype for microglia isolated from adult brain of Lewis and Brown Norway rats as CD45^low^CD11b/c^+^, as distinct from all blood-derived leukocytes being CD45^high^ ^33,34^. To determine heterogeneity of immune cells infiltrating C6 gliomas, we identified microglia as CD11b^+^CD45^low^ cells, peripheral macrophages as CD11b^+^CD45^high^; and leukocytes as CD11b^-^CD45^high^ (Fig. 1A, the lower panel shows a gating strategy). We found 13% of microglia in sham-operated animals; macrophages and leukocytes in brain hemispheres of naïve rats were not detected. At the day 21^st^ after implantation, the percentage of microglia increased to 28% in tumor-bearing brains, while increases in macrophages and leukocytes were 4% and 20%, respectively (Fig. 1A).

To ascertain kinetics of immune cell accumulation during tumor progression, we determined a relative content of glioma-infiltrating immune cells at day 8th, 14th and 21^st^ post-implantation. The abundance of microglia in tumor-bearing hemispheres did not changed at the 8^th^ day after implantation and the significant increase was detected at the 14^th^ and 21^st^ day after cell implantation (Fig. 1B). We found the significantly increased infiltration of peripheral macrophages and leukocytes C6 glioma-bearing brains at days 14^th^ and 21^st^ as compared to sham-operated animals. Surgery itself did not statistically change the immune cell content when naïve and sham-operated groups were compared (Supplementary Fig. S1). Using double immunostaining for Iba1 and Arg1 (a pro-tumorigenic phenotype marker) we demonstrated that a majority of GAMs accumulating in glioma-bearing hemispheres express Arg1, which suggests their pro-tumorigenic, immunosuppressive activation (Fig. 1C).

### Characterization of global transcriptional responses in C6 glioma-bearing hemispheres

**Figure 2:**
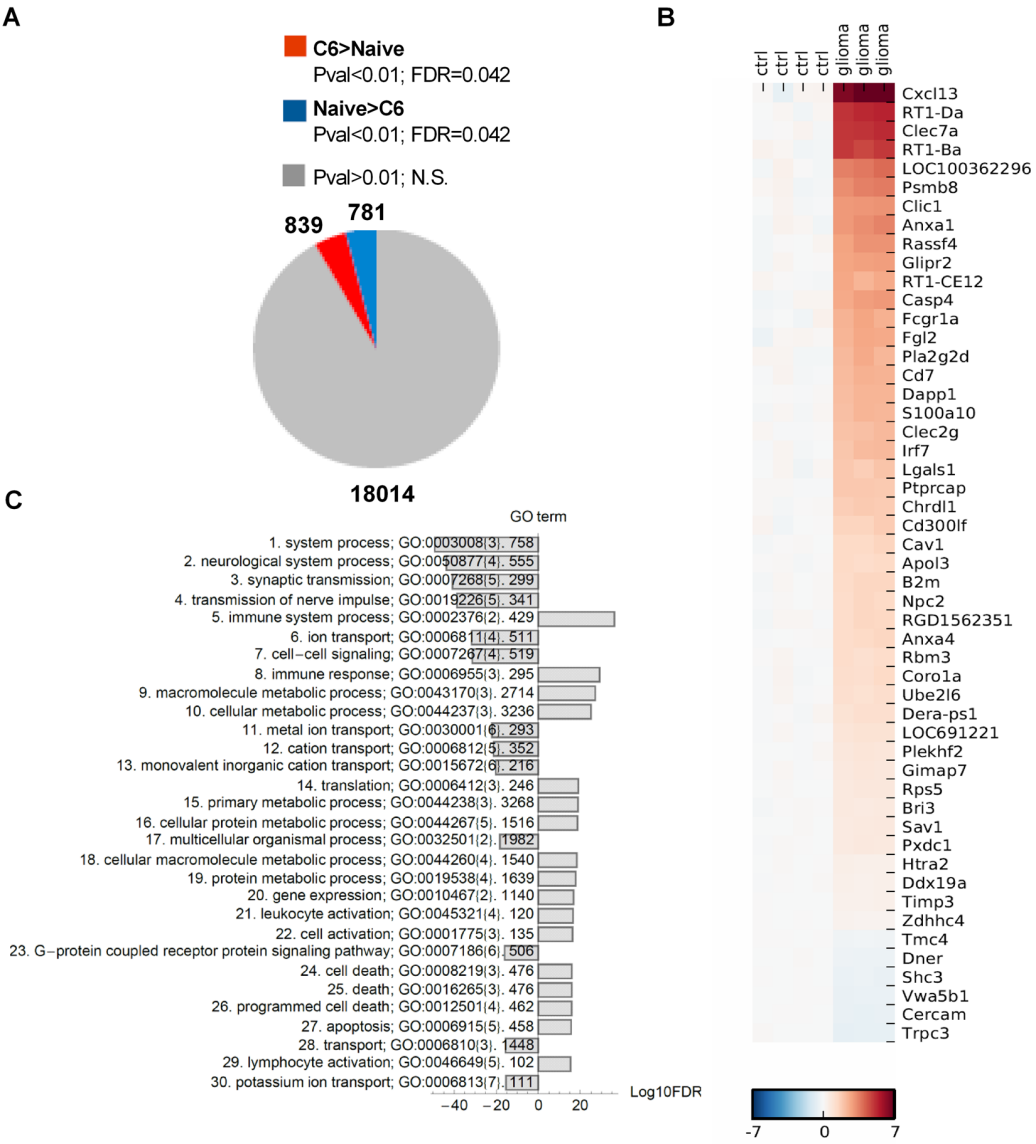
Global gene expression profiling of rat C6 gliomas shows overrepresentation of the immune activation signature genes. (**A**) Pie chart diagram shows numbers of significantly changed genes. Global gene expression in brains of naïve rats (n = 4) and glioma bearing brains (n = 3) was determined using Affymetrix microarrays hybridization. (**B**) For the heatmap generation, a log2 expression profile of each gene (each row) was centred on the average value in the control group, so the color indicates the log2(ratio) change in expression relative to the control and fold. (**C**) Gene ontology (GO) enrichment analysis was performed on all >2 fold upregulated genes that clustered into the glioma-regulated modules to identify overrepresented GO terms. GO terms were grouped according to a number of genes in given category.

We determined transcriptomic profiles of experimental C6 gliomas using isolated total RNA from naïve brains and tumor-bearing brain hemispheres. Global transcriptomic analysis was performed by Affymetrix microarray hybridization. The uniform alpha level for the t-test p < 0.01 was used to identify genes affected by the glioma (this corresponds to an FDR < 0.042 for the change in gene expression). We found that the expression of 1620 genes significantly changed in glioma-bearing brain compared to naïve controls (Fig. 2A). According to the co-expression pattern, we clustered genes into different modules: 781 genes were significantly downregulated (blue) and 839 genes were upregulated (red) (Fig. 2A). The list of 50 genes with the strongest regulation in glioma-bearing hemispheres compared to controls is presented in the Fig. 2B. This group of genes included *CXCL13* encoding a chemokine (C-X-C motif) ligand 13, *Irf7* encoding interferon responsive factor 7 and genes that belongs to RT1 family: *RT1-Da*, *RT-Ba*, *RT1-CE12*. The sub-region *RT1*.*B/D* codes for a rat major histocompatibility complex (MHC) class II^35^, *RT1-CE12* codes for RT1 MHC class I protein. Among upregulated genes we found: *Anxa1* and *Anxa4* coding for Annexin A1 and Annexin A4, *Clec7a* coding for C-type lectin domain family 7 member A which functions as a pattern receptor specific for beta-1,3-linked and beta-1,6-linked glucans, *Clic1* coding for chloride intracellular channel 1, *Psmb8* coding for immunoproteasome subunit, β type 8 related to autoimmunity (Fig. 2B).

Next, we performed the analysis of the functional content of regulated genes grouped in a “biological process”. Gene Ontology (GO) terms associated with changes in gene expression were identified using RankGOstat^31^. The list of 30 overrepresented GO terms with the most significant regulation in glioma-bearing hemispheres compared to controls is presented in the Fig. 2C. The most overrepresented GO terms that characterize transcriptomic responses in the glioma-bearing hemispheres can be grouped into categories such as: neurological system process, metabolic process, cell death and immune responses. Notably, there was the group of the upregulated genes related to cell death and apoptosis, which likely reflects processes of neuronal cell death in glioma-bearing hemispheres. This is consistent with the prominent downregulation of gene expression related to neurological system processes, synaptic and nerve impulse transmission (Fig. 2C).

### Transcriptomic changes in microglia isolated from C6 glioma-bearing hemispheres

**Figure 3:**
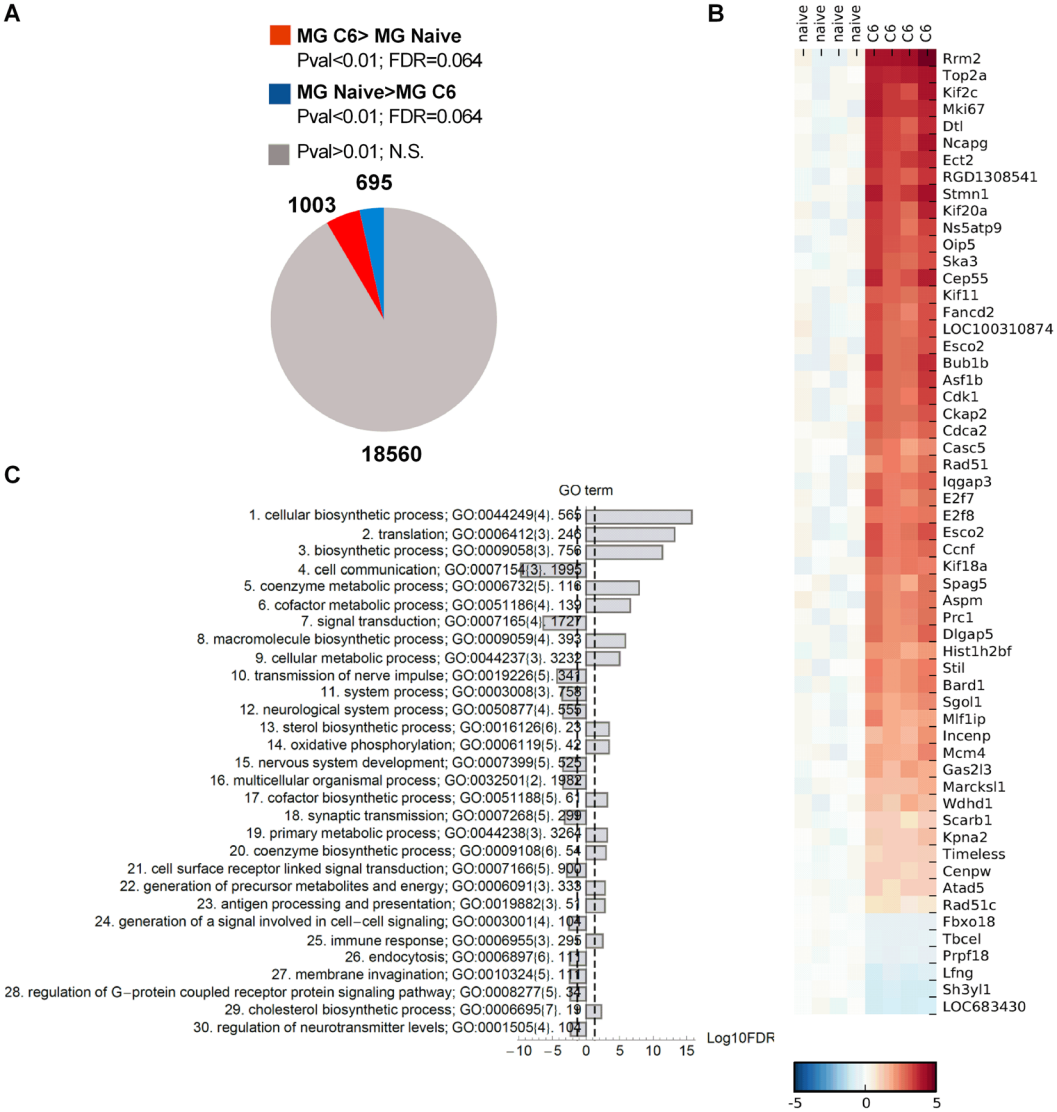
Gene expression profiling of C6 gliomas infiltrating microglia shows markers of the pro-invasive, immunosuppressive activation. (**A**) Pie chart diagram shows numbers of significantly changed genes. Global gene expression in microglia (CD11b^+^CD45^low^) immunosorted from brains of naïve rats (n = 4) and glioma-bearing brains (n = 4) was determined using Affimetrix microarrays hybridization. (**B**) For the heatmap generation, a log2 expression profile of each gene (each row) was centred on the average value in the control group, so the color indicates the log2(ratio) change in expression relative to the control. Gene ontology (GO) enrichment analysis was performed on all >2 fold upregulated genes that clustered into the glioma-regulated modules to identify overrepresented GO terms. GO terms were grouped according to a number of genes in given category.

We sought to determine a functional phenotype of tumor-infiltrating microglia, as these are the first innate immune cells which encounter glioma cells. Microglia (CD11b^+^CD45^low^) were immunosorted by flow cytometry from tumor-bearing hemispheres and naïve animals. To select transcripts induced by the presence of glioma cells, the uniform alpha level for the t-test p < 0.01 was used to identify genes affected by the glioma. This choice corresponded to an FDR < 0.062 for the change induced by glioma. Using a fold change difference of −5 to + 5 and a p-value 0.05 to determine differently expressed genes between naïve and glioma-exposed microglia, we observed totally 1698 genes affected by glioma compared with naïve controls, with 695 genes downregulated (blue) and 1003 upregulated (red) (Fig. 3A). We selected 50 genes with the strongest regulation in microglia from glioma-bearing brains compared with controls (Fig. 3B). Among this group the most upregulated were: *Rrm2* coding for ribonucleotide reductase M2, *Top2a* coding for topoisomerase (DNA) II alpha and *Kif2c*, *Kif11*, *KIF20a* coding for kinesin-related motor proteins. Several genes representing common cell cycle associated genes showed the coordinate increases: *E2f7*, *E2f8* (coding for the atypical E2F transcription factors E2F7 and E2F8), *Cdk1* (coding for cyclin-dependent kinase 1), *Cdca2* (coding for the phosphatase scaffold RepoMan/Cdca2)^36,37^.

The functional analysis of the regulated genes indicated GO terms such as cellular biosynthetic process, translation and mitosis, biosynthetic and metabolic processes, immune response as overrepresented in microglia isolated from glioma-bearing hemispheres compared to controls. In contrast, we found cell-cell signaling and cell surface receptor signal transduction, G-protein coupled receptor signaling, endocytosis and membrane invagination, regulation of neurotransmitter levels among the downregulated GO terms (Fig. 3C).

### Microglia infiltrating glioma-bearing hemispheres present a unique activation pattern

**Figure 4:**
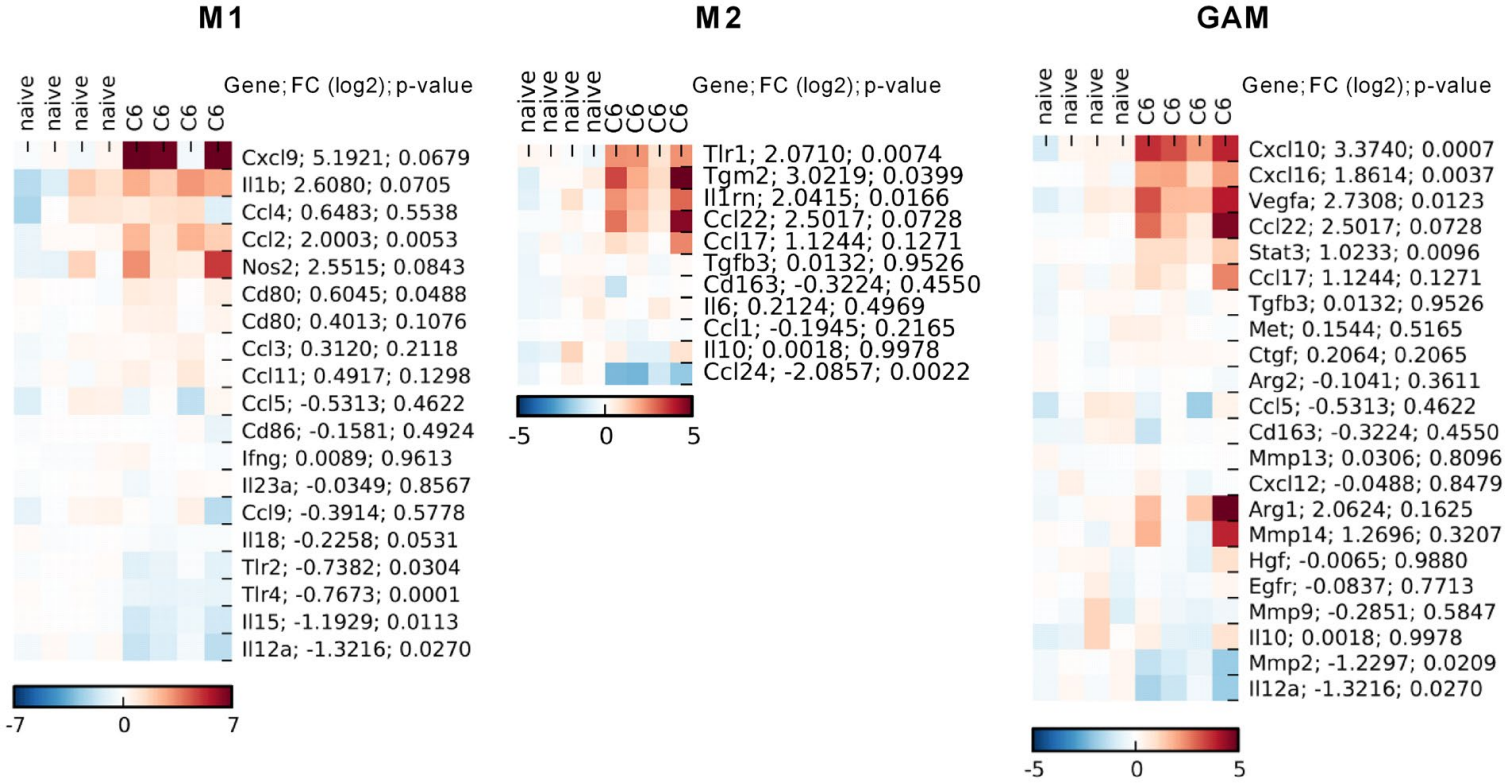
Characterization of expression pattern of known M1/M2 markers in microglia infiltrating C6 gliomas shows upregulation of cell cycle, invasion, immunomodulation and immunosuppression related genes. Global gene expression in microglia (CD11b^+^CD45^low^) immunosorted from brains of naïve rats (n = 4) and glioma-bearing brains (n = 4) was determined using Affymetrix microarrays hybridization. Heatmaps show a log2 expression profile of each gene (each row) which was centred on the average value in the control group, so the color indicates the log2(ratio) change in expression relative to the control; a first column indicates gene name, a second column shows fold change and a third column represents p-value. M1/M2 markers were selected based on guidelines described by Murray *et al*. 2015; GAMs are genes upregulated in CD11b^+^ infiltrating murine GL261 gliomas (Szulzewsky *et al*. 2015).

To define more precisely a type of functional activation in microglia isolated from glioma bearing hemispheres, we used a set of M1/M2 phenotype marker genes^22,38,39^. In microglia sorted from rat C6 gliomas, genes characteristic for M1 activation: *Tlr2*, *Tlr4* and *Il18*, *Cd80*, *Il12a*, *Il15* were significantly downregulated (Fig. 4). The expression of *Cxcl9*, *Nos2* and *IL1β* (M1-specific genes) was increased, but not significantly. *Tlr2* and *Tlr4* code for Toll-like receptor 2 and 4 (pattern-recognition receptors promoting the innate immune defense and inflammation). Interleukins IL-12, IL-15 and IL-18 are known as anti-tumor interleukins^40^. The interleukins IL-12 and IL-23 are pro-inflammatory cytokines produced by dendritic cells, macrophages and fibroblasts in response to microbial infections^41^. IL-12 acts as a mediator of CD4^+^ T cell differentiation towards interferon γ producing Th1 cells^42^. IL-23 amplifies and maintains a Th17 subset of CD4^+^ T cells^43^.

Among genes characteristic for M2 activation and previous described as induced in GAMs, we found the expression of *Tgm2*, *Il1rn*, *Cxcl10*, *Cxcl16*, *Ccl2*, *Ccl17*, *Ccl22*, *Vegfa*, *Stat3*, *Arg1* and *Mmp14* upregulated. The expression of *Ccl24*, *Il12a and Mmp2* was downregulated (Fig. 4). Tgm2 encodes transglutaminase 2, which catalyzes the cross-linking of proteins, stabilizes the extracellular matrix, and is upregulated in fibrosis and cancer metastasis^44^. CCL17 and CCL22 act via CC chemokine receptor 4 (CCR4) predominantly expressed by Th2 cells and Tregs^45^. CXCL16 is a ligand of the chemokine receptor CXCR6 involved in regulating cancer invasion and metastasis^46^. CXCL10 via its receptor CXCR3 (C-X-C motif receptor 3) mediated chemotaxis different innate immune cells amplifying accumulation of myeloid cells^47,48^. Altogether, transcriptomic data suggest that glioma-infiltrating microglia express chemokines and cytokines that regulate extracellular matrix, angiogenesis, chemotaxis and trafficking of different innate and regulatory immune cells. At the same time, these cells display the reduced expression of inflammation mediators and immunomodulators, which may contribute to the immunosuppressive microenvironment.

### Dissecting immune cell heterogeneity in C6 gliomas

**Figure 5:**
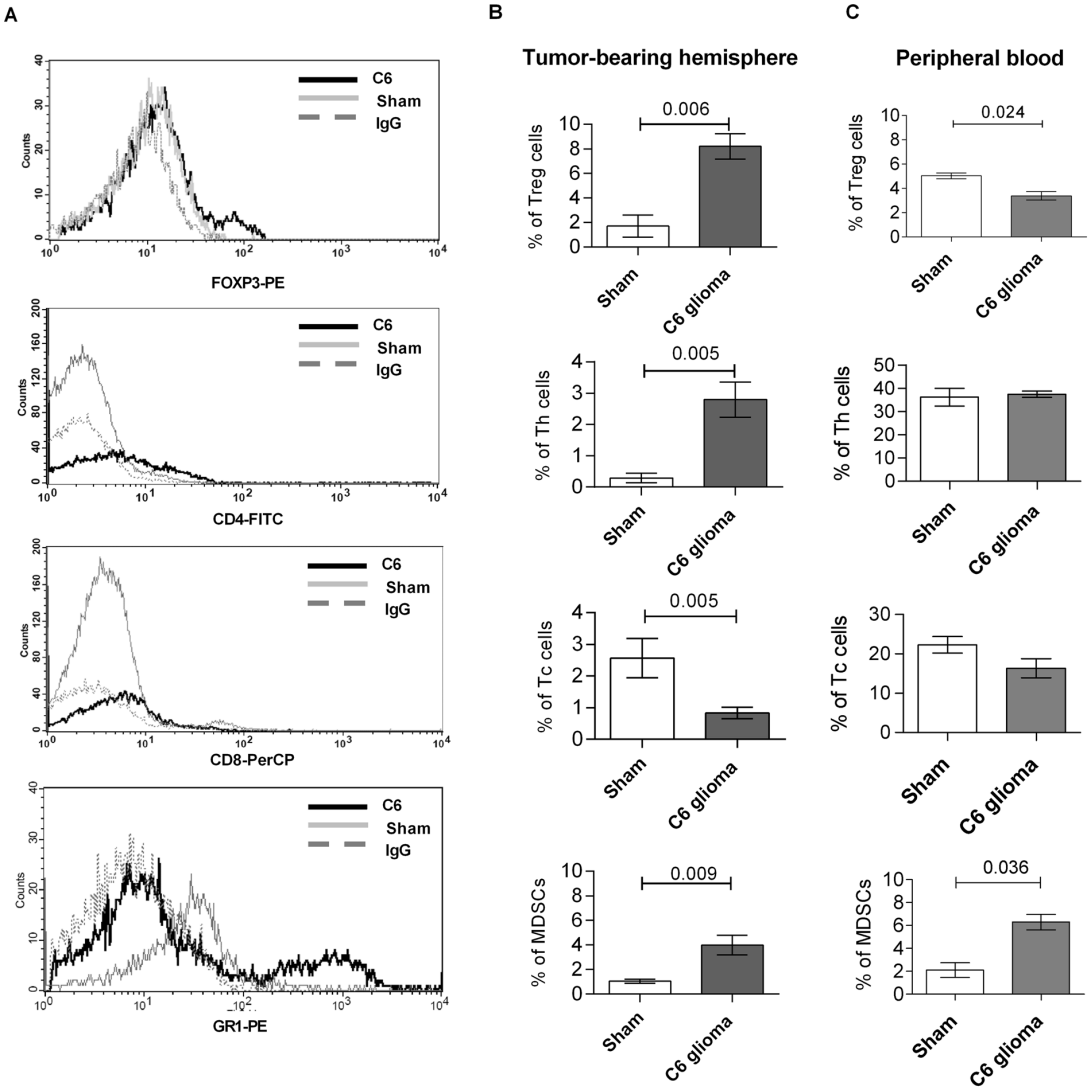
Accumulation of MDSCs and T cell subpopulations in rat C6 gliomas. (**A**) Representative graphs show the analysis of percentages of T regulatory (FOXP3^+^), T helper (CD4^+^), T cytotoxic (CD8^+^) lymphocytes and MDSCs (GR1^+^ within CD11b^+^ cell population) in tumor-bearing hemispheres at the 21st day after C6 glioma cell implantation. Histograms from different subsets isolated from gliomas (dark lines), sham groups (light lines) and IgG control (dotted lines). (**B**) Quantification of percentage of specific cells among all counted cells isolated from sham-operated and tumor-bearing hemispheres at the 21st day after C6 glioma cell implantation (N ≥ 6 per group). (**C**) Quantification of percentage of specific cells among all counted cells isolated from peripheral blood of sham-operated and tumor-bearing animals (N ≥ 6 per group).

Detection of upregulated expression of immunosuppression-related genes in microglia isolated from gliomas prompted us to study infiltration of lymphocytes and MDSCs in gliomas. Specific subpopulations of T cells in sham or tumor-bearing hemispheres were detected by staining with antibodies against CD8, CD4 and intracellular FOXP3; MDSCs were detected by staining with anti-CD11b and anti-Gr1 antibodies. The Fig. 5A shows representative graphs for specific antibodies and isotype controls. T cells were rarely detected in sham-operated brains and reflected cells from circulating blood. If tumor-bearing brains were perfused before FACS analyses, the percentage of Tc was significantly reduced (Supplemental information Fig. S2). Importantly, in glioma-bearing brains we found significantly increases of Treg and Th lymphocytes: 8.20 ± 1.04% (p = 0.006) and 2.80 ± 0.56% (p = 0.005), respectively (Fig. 5B). The increase in Tregs was accompanied by the reduced percentage of Tregs in the peripheral blood, suggesting trafficking of those cells to glioma microenvironment. Percentages of Tc lymphocytes were slightly reduced in tumor-bearing brains as compared to controls (Fig. 5B). Perfusion procedure did not statistically change percentages of Th and Treg in perfused brain tissues, which suggests accumulation of immune cells in the tumor-bearing brain parenchyma. We found the significant increase in the percentage of MDSCs at 21^st^ day after C6 cells implantation both in glioma-bearing brains (1.05 ± 0.175% vs. 3.99 ± 0.81%; p = 0.009) and in the peripheral blood (2.11 ± 0.63 vs. 6.30 ± 0.67%; p = 0.036; respectively) (Fig. 5D). These results show accumulation of immunosuppressive cells: MDSCs, Treg and Th lymphocytes in glioma bearing brains and blood, and demonstrate systemic immunosuppression.

**Figure 6:**
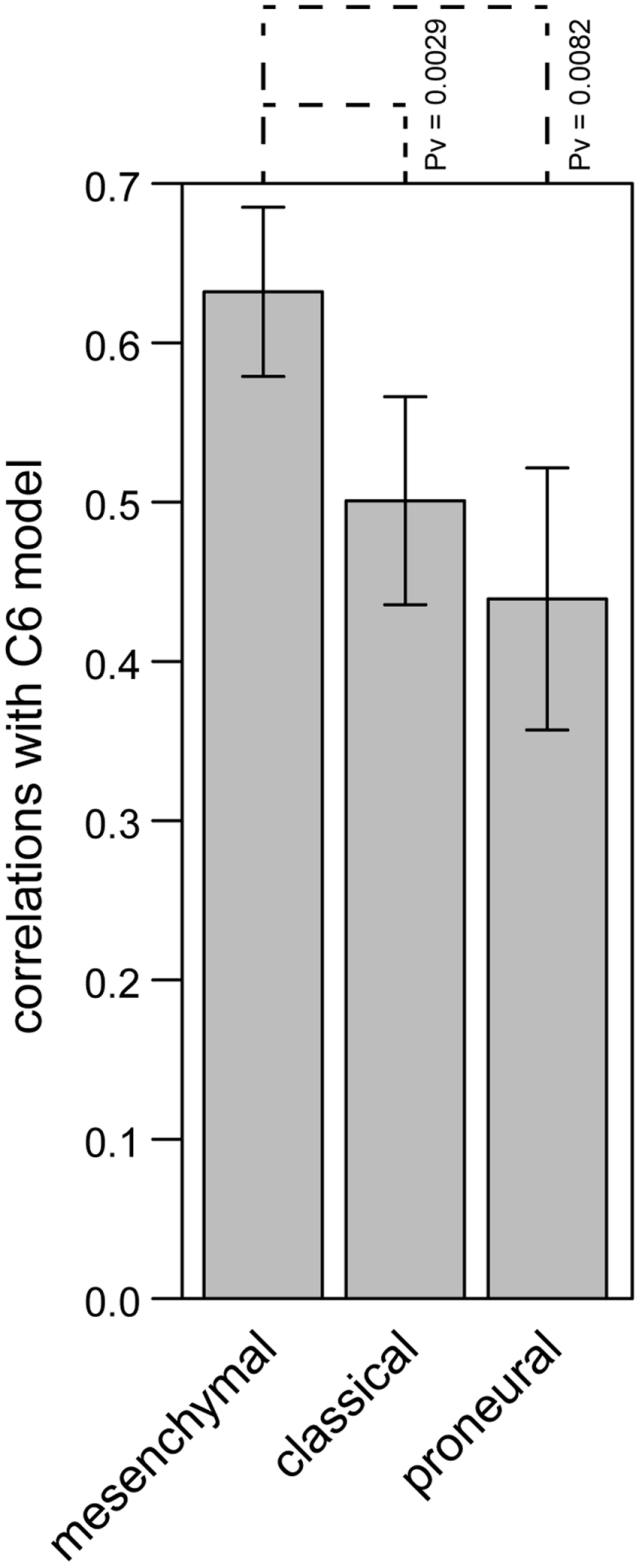
Comparison of gene expression in C6 gliomas and GBM subtypes. Bars represent average correlation coefficients and whiskers show standard deviation (n = 3). The P-values were computed using two-sided Mann-Whitney test.

The detected composition of immune infiltrates and transcriptomic profiles in C6 gliomas indicating systemic immunosuppression and the pro-invasive phenotype of microglia encouraged us to study if transcriptomes of C6 gliomas are comparable to human GBMs. It is known that histopathological features of C6 gliomas show similarity to human GBMs: high proliferation index, diffusive growth, endothelial proliferation and palisading cells around necrotic foci. We sought to check if there is similarity of C6 gliomas to a specific subtype of GBM. RNA-seq profiles (level 3) of GBM and control samples were downloaded from TCGA website. GBM subtypes (Proneural, Classical and Mesenchymal) were assigned using the 840-gene signature published in (^33^, modified) and one-sample Kolmogorov-Smirnoff test. Rat genes were mapped to their human orthologues using 87 release of Ensembl annotation. The list of orthologues contained 660 out of 840 genes included in the signature. Spearman’s correlation coefficients between fold-changes (tumor/normal) of the 660 genes were used to compare C6 gliomas and GBM subtypes (Fig. 6). The highest similarity was found with mesenchymal GBMs.

## Discussion

### Immune cell heterogeneity in rat C6 gliomas

We report the following findings: 1) kinetics and composition of immune infiltrates in rat experimental gliomas show the predominant presence of infiltrating microglia and leukocytes in the glioma microenvironment; 2) the transcriptomic analysis of glioma-bearing hemispheres shows strong upregulation of microglial and immune response genes, while neuronal function genes were downregulated and cell death-related genes were upregulated; 3) the tumor infiltrating microglia acquire the pro-tumorigenic, immunosuppressive phenotype; 4) rat gliomas are infiltrated with MDSCs, Treg and Th to similar extent as human GBMs. Combining these data with known histopathological characteristics, detected similarity of global transcriptomic profiles of C6 gliomas and tumor-infiltrating microglia to those from mesenchymal GBMs, we conclude that C6 gliomas employ similar mechanisms of immune evasion as GBMs. We show predominant accumulation of microglia and leukocytes at days 14 and 21 after implantation of glioma cells. The percentage of macrophages in glioma-bearing brains increased at the same time, but their contribution to the pool of CD11b^+^ cells was relatively low, and these cells accounted for 4–5% of all CD11b^+^ cells. This is in agreement with results of Badie and Schartner, who reported that microglia accounted for 13–34% of the C6 tumor mass, and a smaller proportion of macrophages^49^. Our results show lower contribution of peripheral macrophages than reported previously for murine GL261 gliomas, where percentages of infiltrating microglia and macrophages were similar^39,50,51^. A recent study of head protected, whole body irradiated chimeras with transplanted GFP-bone marrow cells demonstrated that microglia, but not peripheral macrophages, are the main source of mononuclear cells in gliomas^52^. While preliminary studies of the immune content of 9 human malignant gliomas reported the predominance of CD11b^+^CD45^high^macrophages (6.25%), with CD11b^−^CD45^+^ leukocytes (2.48%) and CD45^low^CD11b^+^ microglia (1.65%)^53^, a recent study using the improved FACS method revealed that the main myeloid contributors in human GBMs were MDSCs (~40%) and microglia (~40%), whereas macrophages (~20%) were less frequent^24^. These differences likely stem from methodological differences.

Importantly, we demonstrated accumulation of MDSCs in C6 gliomas. Due to the lack of relevant antibodies, we could not characterize MDSC subpopulations in the rat. MDSCs, which are immature cells of the myeloid linage, are frequently upregulated in tumor patients and experimental animals with different types of cancer, and are known to suppress the adaptive and innate immunity^54–56^. In human GBMs MDSCs (CD33^+^CD11b^+^HLA-DR^−/low^) represented 5.4 ± 1.8% of total cells and the majority of them was described as early-stage, lineage negative (CD14^-^CD15^−^) e-MDSCs, followed by polymononuclear-PMN (CD11b^+^CD14^−^CD15^+^) and macrophage-M (CD11b^+^CD15^-^CD14^+^) subtypes^57^. Another study reported the higher frequency of M- and PMN-MDSCs in peripheral blood of GBM patients compared with healthy donors and correlation between PMN-MDSCs and PD-1 expressing CD4^+^ effector memory T-cells^12^. In murine gliomas, MDSCs accounted for 8.06 ± 0.78% of total cells. In rat C6 gliomas, MDSCs accounted for 4% of cells in tumor-bearing hemispheres and 6% cells in the peripheral blood (Fig. 5).

We found the low percentage of cytotoxic Tc cells, and infiltration of regulatory Th and Treg cells into tumor-bearing hemispheres, which is consistent with the immunosuppressive switch in host immunity observed in human GBMs^28,58–60^. Furthermore, glioma cells and GAMs express *Tgf-β* and *Il-10*, which may contribute to inhibition of T cell activation and proliferation, and stimulate differentiation of naïve T cells into Treg^25^. Gene expression profiling of C6 glioma-bearing hemispheres showed significant overexpression of the previously described Treg markers^21,61^, such as: *Foxp3*, *Ctla-4* (p = 0.049; p = 0.050, respectively) at 21^st^ day after implantation (data not shown). Altogether, our results indicate that immune infiltrates in rat C6 gliomas resemble those in human GBM.

Global gene expression profiling of glioma-bearing hemispheres shows significant changes in the expression of 1620 genes: 781 genes were significantly downregulated and 839 genes were upregulated compared with controls. The analysis of the functional content of regulated genes indicated that GO terms including *Immune responses*, *Cellular biosynthetic process*, *Translation* and *Mitosis* were overrepresented in glioma-bearing hemispheres compared to controls. Genes from categories described as *Immune system process* and *Immune response* are upregulated and reflect activation of GAMs. Adult human GBMs with the shortest survival have significant enrichment of microglia/macrophage-related genes; macrophage recruitment and activation genes are strongly associated with survival^62^. Recent immunostaining and RNA-Seq data showed that accumulation of GAMs and their transcriptomic signatures correspond to the reduced patient survival^63^. Studies performed across 11 tumor types demonstrated that the macrophage signature predicts worse survival in GBMs^13^.

Among downregulated GO terms we found those related to C*ell-cell signaling and Cell surface receptor signal transduction*, *Synaptic and Nerve impulse transmission*, *G-protein coupled receptor signaling*, *Endocytosis*, *Membrane invagination*, *Regulation of neurotransmitter levels* (Fig. 3C). This is consistent with neuronal death occurring during glioma expansion. The peritumoral excitotoxic neuronal cell loss is caused by GBM-secreted glutamate acting through N-methyl-D aspartate receptors^64,65^. Altogether, comparison of global gene expression profiles of C6 gliomas with those reported for human GBMs shows significant similarity of global transcriptomic responses.

### Transcriptomic profiles of C6 glioma-infiltrating microglia

Data on functional phenotypes of GAMs infiltrating human and murine gliomas are conflicting and indicate either a mixture of M1/M2 markers^23,39^ or M0 phenotype^24^. We focused on profiling of gene expression in microglia (CD11b^+^CD45^low^ cells), the early infiltrating and most abundant, immune population in C6 gliomas. Among the most upregulated mRNAs were: *Rrm2* coding for ribonucleotide reductase M2, *Top2a* coding for topoisomerase (DNA) II alpha and *Kif2c*, *Kif11*, *KIF20a* coding for kinesin-related motor protein. RRM2 participates in most of biosynthetic process ensuring accurate DNA replication and repair^66^. High expression of RRM2 reflects enhanced DNA replication and was correlated with poor prognosis of non-CNS cancers: hepatocellular carcinoma, breast and prostate cancer^67,68^. TOP2A is involved in human glioma response to irradiation and regulation of apoptosis^69^. Kinesins Kif2c, Kif11, KIF20a play important roles in mitosis and meiosis^70,71^, and their increase likely reflects microglial proliferation and motility. *Stmn1* encodes Stathmin 1, overexpressed in proliferating hematopoietic cells, myelodysplastic syndrome and acute leukemia cells^72^ and involved in U251 glioma proliferation *in vitro* and tumor growth *in vivo* ^73^. E2F7/8 are transcriptional repressors^74–76^. Currently, roles of those E2Fs in microglia activation are unknown, but E2F7 was reported to repress cell cycle genes and control S-phase progression^77^. Moreover, E2F7/8 directly bind and together with the hypoxia inducible factor 1 (HIF1) stimulate the vascular endothelial growth factor A (*VEGFA*) promoter activity^78^. This is consistent with the prominent role of microglia in tumor angiogenesis^79^. The microglial signature reflects cell cycle regulation and modulation of angiogenesis.

More detailed analysis of gene expression profiles confirmed the pro-tumorigenic phenotype of glioma-infiltrating microglia. Genes characteristic for M1 inflammatory activation: *Tlr2*, *Tlr4*, *Il18*, *Il12a*, *Il15*, *Cd80*, *Ccl24* were significantly downregulated in microglia sorted from rat C6 gliomas (Fig. 4).*Tlr2* and *Tlr4* codes for TLR 2 and 4, pattern-recognition receptors involved in promoting innate immune defense and inflammation. Interleukins IL-12, IL-15 and IL-18 are known as anti-tumor interleukins^40^. The interleukins IL-12 and IL-23 are pro-inflammatory cytokines produced by dendritic cells, macrophages and fibroblasts in response to microbial infections^41^. IL-12 acts as a mediator of CD4^+^ T cell differentiation towards interferon γ producing T_H_1 cells^42^. IL-23 amplifies and maintains T_H_17 subset of CD4^+^ T cells^43^. In preclinical studies of human malignant gliomas IL-12 promoted an immune response against tumor cells, increased the antitumor effect and produced an anti-angiogenic effect^80^. In murine GL261-luc gliomas an IL-15 superagonist complex (IL-15N72D:IL-15RαSu-Fc) as a single treatment or in combination with anti-PD-1 antibody, induced a robust anti-tumor immune response resulting in prolonged survival or tumor remission^81^. The reduced expression of interleukins IL-12, IL-15 and IL-18 could be a part of the immunosuppressive polarization of microglia.

Among genes characteristic for M2 activation and previously described as induced in GAMs, we found the upregulated expression of *Tgm2*, *Il1rn*, *Cxcl10*, *Cxcl16*, *Ccl2*, *Ccl2*, *Ccl17*, *Ccl22*, *Vegfa*, *Stat3*, *Arg1* and *Mmp14* (Fig. 4). Tgm2 encodes transglutaminase 2 which catalyzes the cross-linking of proteins stabilizes the extracellular matrix^44^. CXCL16 is a ligand of the chemokine receptor CXCR6 involved in regulating cancer invasion and metastasis^46^. Two CC chemokine ligands: CCL17 and CCL22 (macrophage-derived chemokine) act via CC chemokine receptor 4 (CCR4) predominantly expressed by Th2 cells and Treg cells^45^. CCL2 and CCL22, are chemokines that attract CCR4-expressing Treg cells^82^. CXCL10 via its C-X-C motif receptor 3 (CXCR3) mediates chemotaxis of different innate immune cells^47^.We demonstrate that glioma infiltrating microglia express numerous chemokines and cytokines that regulate extracellular matrix, angiogenesis, chemotaxis and trafficking of different innate and regulatory immune cells, thus amplifying accumulation of myeloid cells and creating the immunosuppressive microenvironment.

Altogether, we found that composition of the immune infiltrates and transcriptional profiles of CD11b + cells in C6 gliomas resemble the immune infiltrates in human mesenchymal GBMs. Overexpression of invasion and immunosuppression-related genes in C6 gliomas and tumor derived microglia suggests that rat C6 gliomas employ similar immune evasion strategies as human GBMs, and represent a good model of an immunocompetent host for preclinical studies. *In vivo* magnetic resonance imaging and angiography studies showed that similarity C6 gliomas to humans malignant gliomas is better than other models^30^. Rat C6 glioma is a commonly used animal brain tumor model in pharmacological studies, thus demonstrating its immmmunological and transcriptional similarity to human mesenchymal GBMs is an important finding. Our study shows the potential great utility of this model in preclinical research because human mesenchymal GBMs show the highest tumor-related inflammation among GBMs and are the most aggressive.

### Disclosure

We did not receive any funding for this work from any of the following organizations: National Institutes of Health (NIH); Wellcome Trust; Howard Hughes Medical Institute (HHMI).

## Electronic supplementary material

Supplemental information
