# Supplementary material for: Immune microenvironment of experimental rat C6 gliomas resembles human glioblastomas
Source: Sci Rep. 2017 Dec 14;7:17556. doi: 10.1038/s41598-017-17752-w (PMC5730558; doi:10.1038/s41598-017-17752-w)
Supplement: Supplementary file 1 — Supplemental information [file 41598_2017_17752_MOESM1_ESM.pdf]

Immune microenvironment of experimental rat C6 gliomas resembles human glioblastomas

Anna Gieryng[1], Dominika Pszczolkowska[1], Katarzyna Bocian[1], Michal Dabrowski[2], Wenson David Rajan[1], Michal Kloss[1], Jakub Mieczkowski[1], Bozena Kaminska[1]

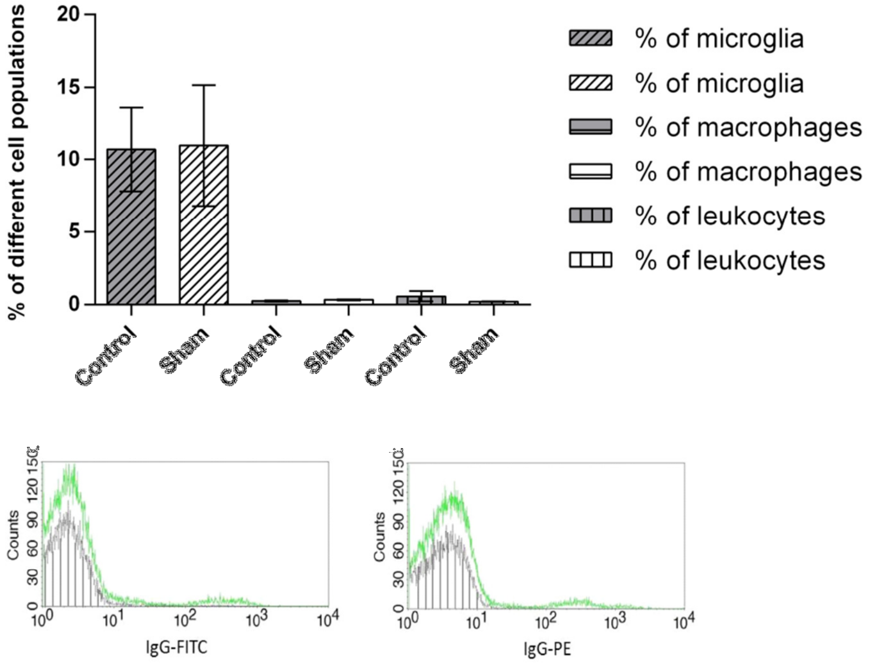

Figure S1. Surgery does not induce significant accumulation of immune cells in rat brain hemispheres

A. The brain tissue samples from naïve controls or sham-operated animals were sacrificed at 21st day and processed to a single cell suspension immediately after brain isolation. Double staining with FITC anti-Rat CD11b and PE anti-Rat CD45 was performed. Results are presented as mean ± SEM (N≥4 per group; t test followed by Mann Whitney U test was performed). **B.** Representative graphs showing specificity of staining with FITC Anti-Rat CD11b IgG (green) and FITC Mouse IgA Kappa Isotype Control (gray) (the left panel), PE Mouse Anti-Rat CD45 (green) and PE Mouse IgG1 Kappa Isotype Control (gray) (the right panel).

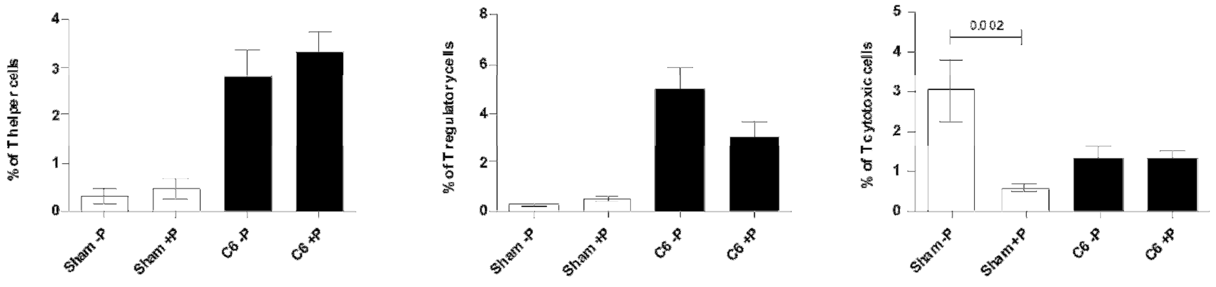

Figure S2. The effects of perfusion on accumulation of T cells in rat brain hemispheres

The brain tissue samples from sham-operated or tumor-bearing animals was isolated at 21st day and processed to a single cell suspension immediately after brain isolation. Some animals were perfused with the use of cold PBS buffer before brain tissue isolation and homogenization (the Sham+P or C6+P groups), in the Sham–P or C6-P groups – brain tissues were isolated and processed immediately. Perfusion did not affect the content of T regulatory and T helper populations in sham and glioma bearing brains. The significantly higher percentage of T cytotoxic cells was observed in brain tissue samples obtained from sham animals processed without perfusion that suggests capturing of some T cells in brain vessels. The perfusion with cold PBS did not affect the content of Tc cells detected in tumor-bearing hemispheres.
